# DNA Polymerase Gamma in Mitochondrial DNA Replication and Repair

**DOI:** 10.1100/tsw.2003.09

**Published:** 2003-03-17

**Authors:** William C. Copeland, Matthew J. Longley

**Affiliations:** Laboratory of Molecular Genetics, National Institute of Environmental Health Sciences, P.O. Box 12233, Research Triangle Park, NC 27709, USA

**Keywords:** mitochondria, DNA polymerase, DNA replication, DNA repair, antiviral nucleoside analogs, aging

## Abstract

Mutations in mitochondrial DNA (mtDNA) are associated with aging, and they can cause tissue degeneration and neuromuscular pathologies known as mitochondrial diseases. Because DNA polymerase γ (pol γ) is the enzyme responsible for replication and repair of mitochondrial DNA, the burden of faithful duplication of mitochondrial DNA, both in preventing spontaneous errors and in DNA repair synthesis, falls on pol γ. Investigating the biological functions of pol γ and its inhibitors aids our understanding of the sources of mtDNA mutations. In animal cells, pol γ is composed of two subunits, a larger catalytic subunit of 125–140 kDa and second subunit of 35–55 kDa. The catalytic subunit contains DNA polymerase activity, 3’-5’ exonuclease activity, and a 5’-dRP lyase activity. The accessory subunit is required for highly processive DNA synthesis and increases the affinity of pol gamma to the DNA.

## INTRODUCTION

DNA polymerase γ is the enzyme responsible for replication of mitochondrial DNA in eukaryotic cells. With the exception of the trypanosomatid parasite[1,2], pol γ is the sole DNA polymerase found in mitochondria, and as such, pol γ must participate in all DNA replication and repair processes. Mitochondria were first visualized as discrete organelles by light microscopy in 1840. However, isolation of intact mitochondria had to wait until zonal centrifugation methods were developed in 1948. In the early 1960s it was determined that these cytoplasmic organelles contain their own DNA. The DNA sequence of human mtDNA was determined in 1981[3] and gene products were assigned by 1985, making mtDNA the first component of the human genome to be fully sequenced. A novel RNA-dependent DNA polymerase activity in eukaryotic cells was first reported in 1970[4,5], and this activity differed from viral reverse transcriptases in that it failed to utilize natural RNA as a substrate[6,7]. The first evidence that the new activity was distinct from pol α and pol β came from column fractionation of Hela cells in 1972[8]. By 1975 this polymerase was designated officially as pol γ, although the cellular function was still elusive[9]. Pol γ was shown to be present in a wide variety of eukaryotic cells, reviewed in (6) and in 1977 pol γ was shown to be present in mitochondria[10]. Evidence was obtained for the functional role of pol γ in mitochondria in a study of isolated brain synaptosomes[11]. Later, disruption of the yeast pol γ gene (MIP1)[12] and inhibition of mtDNA replication in mitochondrial extracts with antibodies raised against pol γ[13] provided further evidence for the role of pol γ.

## POL γ STRUCTURE AND SUBUNIT COMPONENTS

Since its first identification as a distinct DNA polymerase, the purification and biochemical characterization of pol γ has been hampered by proteolysis, oxidative damage, and naturally low abundance. Mitochondrial DNA accounts for approximately 1% of the cellular DNA, and pol γ activity comprises only 1–5% of the total cellular DNA polymerase activity[6,7]. A search of the literature prior to the mid 1980s reveals molecular weight values reported for pol γ ranging from 47–330 kDa[6]. We now know that pol γ is composed of two distinct subunits in animal cells and only one polypeptide in yeast. Animal cell pol γ was shown unequivocally in *Drosophila melanogaster* by Wernette and Kaguni[14] to contain two subunits of 125 and 35 kDa. Pol γ from *Xenopus laevis* contains two subunits of 140 and 50 kDa[15], and an initial report on human Hela cell pol γ identified 140 and 54 kDa polypeptides in the most purified fraction[16]. Originally cloned from *Saccharomyces cerevisiae*[17], the coding sequences for the larger, catalytic subunit have been isolated from human, mouse, chicken, *X. laevis*, *D. melanogaster*, *Schizosaccharomyces pombe*, and *Pychia pastoris*[18,19,20,21]. The predicted sizes for these proteins range from 115 kDa for *S. pombe* to 143 kDa for *S. cerevisiae*, and all the genes contain conserved sequence motifs for polymerase and 3'→5' exonuclease functions. Based on extensive homology alignments[22], pol γ has been grouped with *Escherichia coli* pol I in the Family A DNA polymerase class.

The gene for the smaller subunit from *Drosophila* was first isolated by Kaguni and colleagues[23]. A BLAST search of the *Drosophila* polypeptide sequence identified a partial cDNA clone of the human pol γ accessory subunit[23]. Amino acid alignment of the *Drosophila*, human, and *X. laevis* accessory subunits revealed significant homology to class II aminoacyl-tRNA synthetases, although the ATP binding site and the anticodon binding site are impaired[24]. The mouse pol γ accessory subunit was crystallized as a dimer and the crystal structure indicated structural similarity to glycyl-tRNA synthetase[25]. The human accessory subunit is a 55-kDa protein and is required for highly processive DNA synthesis[26,27,28]. The p55 accessory subunit forms a high affinity, salt-stable complex with p140, and gel filtration and sedimentation analyses reveal a 190-kDa complex indicative of a native heterodimer[26]. Reconstitution of p140•p55 raises the salt optimum of p140, stimulates the polymerase and exonuclease activities, and increases the processivity of the enzyme by several hundredfold. Similar to p140, isolated p55 binds DNA with moderate strength and specificity for double-stranded primer-template DNA. However, the p140•p55 complex has a surprisingly high affinity for DNA, and kinetic analyses indicate p55 enhances the affinity of p140 for primer-termini by two orders of magnitude. Thus the enhanced DNA binding caused by p55 is the basis for the salt tolerance and high processivity characteristic of pol γ.

## ROLE OF POL $\gamma$ IN MITOCHONDRIAL DNA REPLICATION

Mitochondrial DNA is replicated in an asymmetric fashion where the L strand is primed by transcription through the H strand origin within the D-loop[29,30]. After two thirds of the nascent H strand is replicated, the L strand origin is exposed, allowing initiation of nascent L strand synthesis. Mitochondrial DNA is replicated by an assembly of proteins and enzymes including pol $\gamma$, single-stranded DNA binding protein (mtSSB), DNA helicase, and a number of accessory proteins and transcription factors[29,30] (Table 1). The cDNA for the single-stranded DNA binding protein that functions in mitochondria has been cloned and predicts a molecular weight of 15 kDa for each monomer[31]. The human mtSSB is 31% identical to *E. coli* SSB. A replicative helicase was identified last year as encoded by the Twinkle gene[32], and a topoisomerase I gene has been identified to function in the mitochondria[33]. Fig. 1 depicts a cartoon of a mtDNA replication intermediate, and Table 1 lists the nuclear gene products required for mtDNA replication.

**TABLE 1**
**Known Nuclear Gene Products Required for mtDNA Replication/Repair***

| **Enzyme** | **Size** | **Human Chromosome** |
|---|---|---|
| DNA polymerase □ | | |
| POLG | 140 kDa | 15q25 |
| POLG2 | 55 kDa | 17q23-24 |
| Single-stranded DNA binding protein | 15 kDa | 7q34 |
| Helicase | | |
| Twinkle gene | 77 kDa | 10q24 |
| RNA transcription | | |
| Core RNA Polymerase | 150 kDa | 19q13.3 |
| mtTFA | 24 kDa | 10q21 |
| mtTFB | 40 kDa | 6q25.1-q25.3 |
| RNase MRP RNA | 275-285-nt RNA | 9p13 |
| Topoisomerases | | |
| Topo I | 67 kDa | 8q24.3 |
| Ligase | | |
| DNA ligase III | 96 kDa | 17q11.2-12 |
| | | |
| **Repair Genes:** | | |
| Glycosylases | | |
| UDG | 27.5 kDa | 12q23-q24.1 |
| OGG1 | 38 kDa | 3p26.2 |
| NTH1 | 34 kDa | 16p13.3 |
| MYH1 | 60 kDa | 1p34.3-p32.1 |
| AP endonucleases | | |
| APE2 | 57 kDa | Xp11.22 |
| Endo G | 30 kDa | 9q34.1 |

*Human genes for mitochondrial primase, RNase H, or FEN1 have not been identified. These activities are thought to be required for mtDNA replication and/or repair.

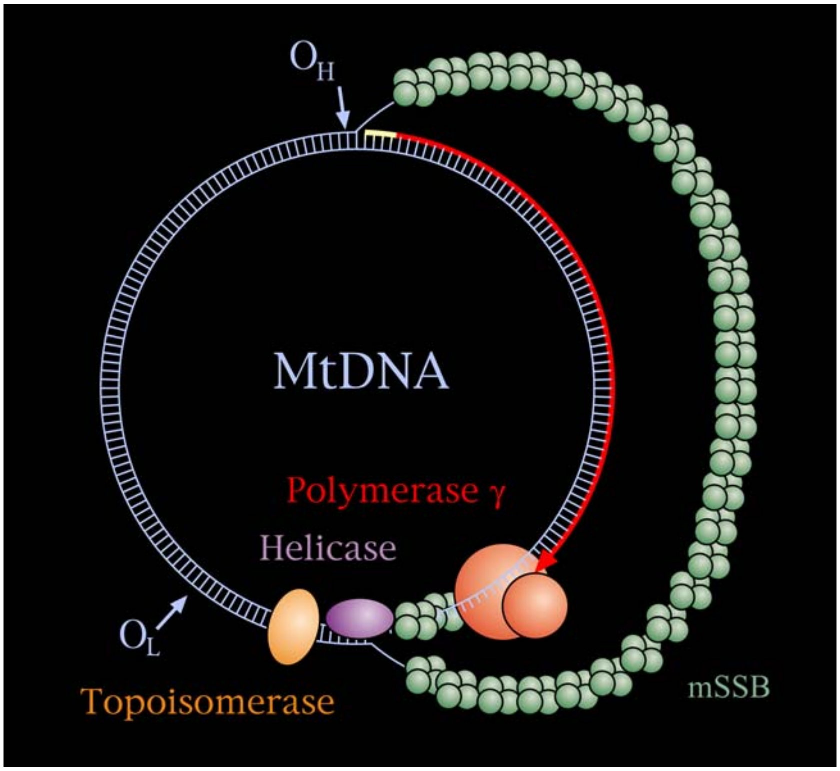

**FIGURE 1.** Schematic diagram of the mitochondrial DNA replication intermediate, showing the critical proteins required for DNA replication. MtDNA replication is initiated at the heavy strand origin (OriH) by transcription within the displacement loop by the mitochondrial RNA polymerase. The nascent transcript is processed by the MRP RNase to form a primer that is elongated by DNA pol γ. When approximately two thirds of the nascent heavy strand has been elongated, OriL is exposed, and replication of the light strand is initiated. The growing displacement loop from H strand synthesis is coated by the mitochondrial single-stranded DNA binding protein. The genes for the proteins required in mtDNA replication are listed in Table 1.

Pol γ is unusual in its ability to utilize a wide variety of template-primers (specifically homopolymeric RNA), and the enzyme also uniquely displays a low Km (<1 μM) for dNTPs. Pol γ is aphidicolin resistant, salt insensitive, and strongly inhibited by dideoxynucleotides. Although the single p140 catalytic subunit of pol γ is **sensitive to N-ethylmaleimide-sensitive**[NEM][34], both the native and recombinant forms of the p140•p55 complex display nearly complete resistance to NEM up to 1 mM[26]. In marked contrast, the single catalytic subunit is inhibited to 50% with less than 0.1 mM NEM and >90% inhibited at 0.5 mM NEM[35]. Thus, the p55 accessory subunit protects the catalytic subunit from NEM inhibition by over 100-fold. DNA polymerase γ is unique among the cellular replicative DNA polymerases in that it is highly sensitive to inhibition by anti-HIV nucleoside analogs, such as AZT, dideoxynucleotides, and other nucleoside analogs[36,37,38,39,40,41,42,43,44,45,46]. These drugs inhibit mitochondrial DNA replication and deplete the organelles of mtDNA. The resulting mitochondrial toxicity mimics mitochondrial genetic diseases and induces similar clinical syndromes, including ragged-red muscle fibers. The cause of dideoxynucleoside sensitivity has been attributed to a single tyrosine in motif B (Y951 in human) of pol γ[34]. This tyrosine is invariant in all of the pol γ sequences and corresponds to Y526 of T7 DNA polymerase, another member of the Family A DNA polymerases. The sensitivity of T7 DNA polymerase to ddNTPs is due to Y526[47] and the presence of this tyrosine in the γ polymerases is consistent with their sensitivity to ddNTPs. Replacement of this tyrosine residue critical for sugar recognition with phenylalanine in motif B

of the human enzyme reduced dideoxynucleotide inhibition by a factor of 5000 with only minor effects on overall polymerase function[34].

## REGULATION

The regulation of expression of pol γ genes is critical to mtDNA content. Depletion of the MIP1 gene in yeast leads to loss of mtDNA and the formation of petites[12]. In contrast, overexpression of the catalytic subunit in *Drosophila* leads to depletion of mtDNA[48], and mutations in the gene for the accessory subunit in *Drosophila* cause loss of mtDNA and lethality[49]. The nuclear respiratory factor 1 (NRF-1) is a transcription factor that regulates many of the nuclear-encoded mitochondrial proteins needed for oxidative phosphorylation and some components of the mitochondrial transcription machinery. Binding of NRF-1 is regulated by the ATP requirements of the cell. The promoters for the human genes of pol γ catalytic subunit, the accessory subunit (p55), and mtTFA contain DNA binding motifs for NRF-1. Expression of the *Drosophila* pol γ accessory subunit is controlled, in part, by a DNA replication-related element (DRE) that normally regulates genes involved in nuclear DNA replication[50].

Pol γ activity has been detected in yeast rho$^{o}$ cells, which lack mtDNA[12]. Pol γ is expressed and translated in cultured human cell lines either containing or lacking mitochondrial DNA, indicating that the pol γ protein is stable in the absence of mitochondrial DNA[51]. In contrast, the same study demonstrated that the stability of mtTFA was tightly linked to the mtDNA status of the cell[51]. Synthesis of mitochondrial DNA, as estimated by incorporation of BrdU, occurs preferentially in perinuclear mitochondria[52]. However the observation of pol γ in both peripheral and perinuclear mitochondria[51] suggests an additional role for pol γ, such as participation in DNA repair.

## ROLE OF POL γ IN MITOCHONDRIAL DISEASES AND AGING

### Fidelity of DNA Replication

Pol γ purified from chicken embryos or from pig liver mitochondria is accurate *in vitro*, with these enzymes exhibiting error frequencies at a three nucleotide mutational target of $<3.8 \times 10^{-6}$ per nucleotide and $<2.0 \times 10^{-6}$ per nucleotide, respectively[53,54]. Both enzymes contain intrinsic 3' to 5' exonuclease activities that prefer mispaired 3'-termini. Partial inhibition of these exonuclease activities with 20 mM dGMP increases the frequency of errors, suggesting the exonucleases proofread replication errors *in vitro*[53,54,55]. Several lines of evidence indicate the exonuclease contributes to replication fidelity *in vivo*. Disruption of the exonuclease motifs in the yeast MIP1 gene generates a mutator phenotype, as exhibited by a several hundredfold increase in the spontaneous frequency of forming mitochondrial erythromycin-resistant mutants[56]. Expression of exonuclease-deficient pol γ fusion proteins in cultured human cells also resulted in the accumulation of point mutations in mitochondrial DNA[57]. Also, the loss of pol γ's exonuclease function in transgenic mice resulted in the rapid accumulation of point mutations and deletions in cardiac mtDNA, and the mutagenesis was accompanied by cardiomyopathy[58].

The human catalytic subunit of pol γ has high base substitution fidelity that results from high nucleotide selectivity and exonucleolytic proofreading[59]. Pol γ is also relatively accurate for single-base additions and deletions in noniterated and short repetitive sequences. However when copying homopolymeric sequences longer than four nucleotides, pol γ has low frameshift fidelity, suggesting that homopolymeric runs in mtDNA may be particularly prone to frameshift mutation *in vivo* due to replication errors by pol γ. Pol γ also generates base substitutions inferred to result from a primer dislocation mechanism. Inclusion of the 55-kDa accessory subunit, which confers processivity to the pol γ catalytic subunit, decreases frameshift and base substitution fidelity. Kinetic analyses indicate that p55 lowers fidelity of replication by promoting extension of mismatched termini[59].

## Mitochondrial DNA Repair and The Role of Pol γ in Base Excision Repair

Although mitochondria lack nucleotide excision repair[60], mitochondria have the capacity to repair damaged bases[61,62,63,64,65,66,67]. Pinz et al.[68] have reconstituted the minimum complement of repair proteins needed for base excision repair from *X. laevis*, and Bohr and colleagues have isolated specific glycosylases which remove 8-oxo-dG and other damaged bases[61,69,70]. Additionally, the identification of the Msh1 gene suggests mismatch repair may occur in yeast mitochondria[71,72,73].

As the only DNA polymerase present in mitochondria, pol γ is necessarily implicated in all such repair processes. Excision repair of a damaged base requires the concerted activities of a glycosylase to remove the damaged or inappropriate base, a class II AP endonuclease to incise the DNA 5' to the AP site, a lyase activity to remove the 5'-terminal 2-deoxyribose-5-phosphate (dRP) sugar moiety from the downstream DNA, resynthesis, and ligation[74]. DNA repair enzymes that have been isolated from mitochondria include uracil-DNA glycosylase[75], AP endonuclease[76], an 8-hydroxydeoxyguanine specific endonuclease[62], and DNA ligase[68]. Pol γ can participate in uracil-provoked base excision repair reconstituted *in vitro* with purified components, where subsequent to actions of uracil-DNA glycosylase and AP endonuclease, pol γ can fill a single nucleotide gap in the presence of a 5' terminal deoxyribose phosphate (dRP) flap[77]. The removal of the dRP moiety can proceed via simple hydrolysis or by enzyme catalyzed β-elimination[74]. Reconstitution experiments with *X. laevis* mitochondrial proteins localized the dRP lyase function to either the mtDNA ligase or pol γ[68]. The catalytic subunit of human pol γ was subsequently shown to catalyze the release of the dRP residue from incised apurinic/apyrimidinic sites to produce a substrate for DNA ligase[77]. The dRP lyase activity does not require divalent metal ions, and the ability to trap covalent enzyme-DNA complexes with $NaBH_4$ strongly implicates a Schiff base intermediate in a β-elimination reaction mechanism. The dRP lyase reaction proceeds by formation of a covalent enzyme–DNA intermediate that is converted to an enzyme-dRP intermediate following elimination of the DNA[78].

## Defects of Pol γ in Mitochondrial Diseases

Mitochondrial genetic diseases are caused by point mutations and deletions in mitochondrial DNA[79]. Mutations can result from spontaneous errors of replication or from unrepaired chemical damage to DNA, such as oxidation or exposure to UV radiation. To date, two mitochondrial diseases have been attributed to alterations in pol γ: progressive external ophthalmoplegia and Alper's syndrome. Progressive external ophthalmoplegia is a rare disease characterized by the accumulation of point mutations and large deletions in mtDNA. Recently, sequence analysis through the pol γ gene[19] in a Belgian pedigree with dominant PEO identified a heterozygous A to G mutation at codon 955 (Y955C)[80]. Located in the active site of pol γ, Y955 is a highly conserved residue among a wide variety of DNA polymerases, and this residue participates in recognition of the incoming nucleoside triphosphate. Analysis of the Y955C mutant polymerase indicates that it retains wild-type catalytic turnover efficiency but suffers a

45-fold decrease in affinity for dNTPs[81]. The Y955C mutation can increase the mutation rate at a single base pair by over 50-fold[81], and misinsertion events following direct repeat DNA sequences can initiate large deletions in mtDNA. This study represents the first biochemical analysis of the mechanisms by which a mutator DNA polymerase generates the mutations observed in certain mitochondrial diseases. Zeviani and coworkers have recently reported eight new mutations in the pol γ gene sequence that cause heritable PEO, but the functional consequences of these mutations are unknown[82].

Alper's syndrome is a rare but severe heritable, autosomal recessive disease that afflicts young children. Within the first few years of life, patients exhibit progressive, spastic paresis of the extremities and progressive mental deterioration leading to seizures, blindness, deafness, and eventual death. Naviaux reported an Alper's patient with reduced electron transport chain function, dicarboxylic aciduria, fulminant hepatic failure, and lactic acidosis which resulted in death at 42 months[83]. Skeletal muscle biopsy indicated a reduction of mtDNA content to 30% of normal with no detectable pol γ activity[83]. Although a heritable deficiency in mtDNA replication is suggested, the genetic defect(s) causing Alper's syndrome remain unreported.

Defects in other nuclear genes controlling maintenance of mtDNA have also been associated with mitochondrial diseases, including the genes for adenine nucleotide translocator 1 (ANT1) at locus 4q34-35[84], thymidine phosphorylase at locus 22q13.32-qter[85], a putative mitochondrial helicase (Twinkle) at locus 10q24[32], and an unidentified gene at locus 3p14-21[86]. Multiple mutations within the "Twinkle" gene encoding a putative mitochondrial helicase are causally linked to dominant PEO with mtDNA deletions[32], while mutations in the nuclear genes for ANT1 or thymidine phosphorylase also induce pathogenic mutation of mtDNA[84,85], perhaps by unbalancing or reducing the available intramitochondrial pool of deoxynucleoside triphosphates. Nucleotide pool imbalance is known to enhance base substitution errors by pol γ[53,55]. Additionally, Wallace observed mtDNA rearrangement and increased production of reactive oxygen species in the mitochondria of $ANT1^{-/-}$ knockout mice, suggesting pathogenesis results from enhanced oxidative damage to mtDNA[87].

## OXIDATION OF POL γ

The mitochondrial respiratory chain is a source of endogenous reactive oxygen species (ROS), and oxidative modification of biomolecules, including proteins, can alter their normal functions. Since pol γ is associated with DNA within the mitochondrial matrix, this enzyme is subject to oxidation *in vivo* by hydrogen peroxide and iron ions associated with mtDNA. The effect of $H_2O_2$ on the enzymatic activities and DNA binding efficiency of pol γ has been examined[88]. Hydrogen peroxide inhibits the DNA polymerase activity of the p140 subunit and lowers its DNA-binding efficiency. Addition of p55 to the p140 catalytic subunit prior to $H_2O_2$ treatment offers protection from oxidative inactivation. Pol γ can be detected as one of the major oxidized proteins in the mitochondrial matrix, and the degree of oxidation correlates with a decline in polymerase activity. These results suggest that pol γ is a target for oxidative damage by ROS, which may impair mitochondrial DNA replication and repair.

## FUTURE DIRECTIONS

Unlike the SV40 DNA replication model for nuclear DNA replication[89], an *in vitro* assay does not yet exist for mtDNA replication. However, as new replication factors are identified, we grow nearer to reconstituting mtDNA replication *in vitro*. Understanding the genetic causes of mitochondrial diseases has proven useful in identifying nuclear genes involved in mtDNA

replication. The combination of biochemistry and genetics will further help to elucidate new functions and the involvement of unknown genes in mtDNA maintenance.
